# Impact of repeated NeemAzal®-treated blood meals on the fitness of *Anopheles stephensi* mosquitoes

**DOI:** 10.1186/s13071-015-0700-1

**Published:** 2015-02-10

**Authors:** Edson G Dembo, Solomon M Abay, Nisha Dahiya, Johnbull S Ogboi, George K Christophides, Giulio Lupidi, Giuseppina Chianese, Leonardo Lucantoni, Annette Habluetzel

**Affiliations:** School of Pharmacy, University of Camerino, Piazza dei Costanti, 62032 Camerino, MC Italy; School of Medicine, College of Health Sciences, Addis Ababa University, Addis Ababa, Ethiopia; Department of Life Sciences, Imperial College London, London, United Kingdom; Department of Pharmacy, University of Naples Federico II, Via Montesano 49, 80131 Naples, Italy; Current address: Discovery Biology, Eskitis Institute for Drug Discovery, Griffith University, Nathan, 4111 Queensland Australia

**Keywords:** Malaria, Vectors, Neem, Azadirachtin, Transmission-blocking, Anti-vectorial

## Abstract

**Background:**

Herbal remedies are widely used in many malaria endemic countries to treat patients, in particular in the absence of anti-malarial drugs and in some settings to prevent the disease. Herbal medicines may be specifically designed for prophylaxis and/or for blocking malaria transmission to benefit both, the individual consumer and the community at large. Neem represents a good candidate for this purpose due to its inhibitory effects on the parasite stages that cause the clinical manifestations of malaria and on those responsible for infection in the vector. Furthermore, neem secondary metabolites have been shown to interfere with various physiological processes in insect vectors. This study was undertaken to assess the impact of the standardised neem extract NeemAzal® on the fitness of the malaria vector *Anopheles stephensi* following repeated exposure to the product through consecutive blood meals on treated mice.

**Methods:**

Batches of *An. stephensi* mosquitoes were offered 5 consecutive blood meals on female BALB/c mice treated with NeemAzal® at an azadirachtin A concentration of 60, 105 or 150 mg/kg. The blood feeding capacity was estimated by measuring the haematin content of the rectal fluid excreted by the mosquitoes during feeding. The number of eggs laid was estimated by image analysis and their hatchability assessed by direct observations.

**Results:**

A dose and frequency dependent impact of NeemAzal® treatment on the mosquito feeding capacity, oviposition and egg hatchability was demonstrated. In the 150 mg/kg treatment group, the mosquito feeding capacity was reduced by 50% already at the second blood meal and by 50 to 80% in all treatment groups at the fifth blood meal. Consequently, a 50 – 65% reduction in the number of eggs laid per female mosquito was observed after the fifth blood meal in all treatment groups. Similarly, after the fifth treated blood meal exposure, hatchability was found to be reduced by 62% and 70% in the 105 and 150 mg/kg group respectively.

**Conclusions:**

The findings of this study, taken together with the accumulated knowledge on neem open the challenging prospects of designing neem-based formulations as multi-target phytomedicines exhibiting preventive, parasite transmission-blocking as well as anti-vectorial properties.

## Background

Recent global estimates suggest that over the last decade malaria-related mortality has been reduced by about 49% in the WHO African region [1]. The successful reduction of malaria-related mortality is partly due to the deployment of free or subsidised insecticide-treated bed nets (ITNs) and artemisinin-based combination therapy (ACT), as well as indoor residual spray (IRS) implementation at a large scale. These malaria control strategies benefit the individual users and confer significant benefits to the local communities by reducing parasite transmission [2,3] (reviewed in [4]).

However, the currently used control tools may become redundant due to the evolution of resistance, by parasite and vector populations to the few available effective drugs and insecticides. For example, the emerging evidence of mosquito resistance to pyrethroid chemicals used for ITNs impregnation [5-10] and resistance to dichlorodiphenyltrichloroethane (DDT) and other chemicals used in IRS [11] threatens the sustainability of current malaria control interventions. In addition, *Plasmodium falciparum* resistance to artemisinin derivatives [1,12-14], a few years after most governments of endemic regions changed to ACTs as first line treatment for malaria, represents yet another major threat to malaria case management. While other drug combinations with chloroquine, quinine and sulphadoxine pyrimethamine are known to decrease gametocytaemia prevalence and mosquito infectiveness [15-17], recent studies have shown that children treated with ACT combinations remain infective to mosquitoes after day 14 of treatment initiation [18] suggesting a rebound effect of gametocytaemia [19].

These challenges highlight a need for additional tools and new strategies for malaria control and elimination. Scientifically validated herbal remedies, for instance, may offer such a tool and constitute a source for the development of new drugs and/or improved phytomedicines. As shown by the history of anti-malarial drugs, traditional medicinal knowledge on plants is a compelling source for the discovery of potent antimalarial molecules; in fact major anti-malarial drugs such as quinine, artemisinin and their derivatives are products of scientific exploitation of traditional knowledge on the medicinal properties of *Cinchona spp* and *Artemisia annua* respectively.

Anti-malarial remedies are still widely employed and culturally accepted by communities; people use herbs for therapy, prevention [20,21] and for mosquito repellence [22-24]. In several countries standardized herbal preparations are available and are considered for the use as first line therapeutic intervention in the absence of ACTs [25]. Relying on the effects of multiple bio-active compounds, herbal anti-malarial medicines are less prone to induce development of parasite resistance and if applied at a large scale, have the potential to reduce drug pressure for the development of resistance to ACTs [25].

In Mali an anti-malarial phytomedicine based on *Argemone mexicana* has recently been included into the national pharmacopeia as a validated herbal treatment for the management of malaria following a successful randomised controlled trial of the herbal remedy vs artesunate amodiaquine [26]. A “clinical recovery” rate of 89% (without need for a second line treatment) was recorded for *A. mexicana* treated patients compared to 95% for individuals of the artesunate amodiaquine control group [26].

Several plant species have been shown to exhibit anti-malarial properties (reviewed in [27]) but only a few have been found to possess a secondary metabolite profile that supports practical utilization for the development of standardised remedies and improved phytomedicines. As an example, for centuries the neem tree (*Azadirachta indica*) has given rise to various medicinal preparations for the treatment of malaria and other illnesses in many parts of the world [28,29]. The success of neem is derived from its broad spectrum properties against parasites and other infective agents (reviewed in [30]), due in part to its rich sources of limonoids which are highly oxygenated terpenoids with insecticidal, anti-microbial, anti-inflammatory and immuno-modulatory activities among other biological properties [30,31].

Azadirachtins, in particular, exhibit potent insect hormone regulatory properties, interfering with various physiological processes of both larval and adult stages of disease vector species [30]. Studies conducted on the Chagas disease vector *Rhodnius prolixus* have shown that azadirachtin A, taken up orally through blood feeding by the insect*,* caused a reduction in oocyst growth and egg production in a dose dependent manner [32]. Azadirachtin A administered orally in sucrose to *Culex tarsalis* and *C. quinquefasciatus* females suppressed blood feeding, reduced the oviposition rate, the size of the egg raft, the hatching rate and longevity of the treated insects [33]. Studies conducted with commercial azadirachtin A enriched neem formulations (NeemAzal® T/S 1.2 percent EC, Trifolio-M GmbH), evidenced larvicidal and emergence inhibitory effects on *An. stephensi, C. quinquefasciatus* and *Aedes aegypti* larval stages [34].

We previously observed impairment in blood intake, oogenesis and oviposition in *An. stephensi* by a NeemAzal® formulation with an azadirachtin A content of 34%. The effects observed revealed to be dose dependent and were observed both in females having received NeemAzal® in blood through membrane feeding and females that had ingested the product with sucrose solution and were provided subsequently with an untreated blood meal [35]. In a successive study, NeemAzal® was found to completely suppress *Plasmodium berghei* infection in mosquitoes when administrated through a blood meal on gametocytemic mice treated with NeemAzal® at a azadirachtin A concentration of 50 mg/kg [36]. Recently, the transmission blocking effect by NeemAzal® was confirmed in *P. falciparum*. A complete blockade of mosquito infection was demonstrated in *Anopheles coluzzii* females membrane fed on gametocytemic blood from *P. falciparum* infected patients containing NeemAzal® at a dosage of 70 μg/ml, while an 80% prevalence reduction was observed at 50 μg/ml [37]. Examination of midgut smears from *An. stephensi* mosquitoes 20 hrs after having fed on NeemAzal® treated *P. berghei* infected mice indicated that early sporogonic stages were the main targets of the product [36].

Neem extracts also show *in vitro* activity on asexual and sexual blood stages of *P. falciparum* , with gedunin, nimbolide, and nimbinin being the most active constituents [38]. The inhibition of chloroquine- and pyrimethamine-resistant strains also, suggests that neem possesses a different mode of action than that of the two drugs [38]. More recently, a neem fruit extract (EtOAc phase) was found to possess prominent *in vitro* antimalarial activity (IC_50_ ~ 2 μg/ml) that could be attributed to the abundant presence of azadirone, gedunin and neemfruitin A, all of which have the ability to inhibit *P. falciparum* D10 and W2 laboratory strains [39]. Furthermore, the different metabolites present in the extracts could have multiple mechanisms of action, a property that can slow down or negate the development of parasite resistance. Hence, based on its multiple stage anti-plasmodial as well as insect regulatory properties, neem represents a very good candidate for the development of a phytomedicine exhibiting at a time preventive, transmission blocking and mosquitocidal activities.

On the basis of the illustrated knowledge, we developed a novel concept to mirror a real life situation where people regularly take a neem-based phytomedicine and therefore expose *Anopheles* mosquitoes to repeated neem-containing blood meals. Female anophelines are anthropophilic and feed every 2 to 4 days per oviposition cycle, therefore mosquitoes could be exposed to multiple neem-containing blood meals during their lifespan. We therefore, hypothesized that neem-treated blood meals will have cumulative biological effects on *Anopheles* mosquitoes in such a setting which may affect them as a population and impact on their vectorial capacity. This study was designed to characterize the effects of repeated NeemAzal® treatments on *An. stephensi* feeding capacity, fecundity (oviposition rates) and fertility (egg hatchability). Such an impact, together with neem antiplasmodial activity in the vector, may lead to blockage of parasite transmission. If this hypothesis is supported, the design and development of a preventive, transmission blocking and “anti-vectorial” neem formulation, guided by translating the accumulated knowledge on the biological effects of azadirachtin and other neem limonoids, could appear feasible.

## Methods

### Experimental animals

Five to seven days old *An. stephensi* females were used. Mosquito colonies were maintained at 30°C, > 75% relative humidity and 12:12 hrs light to dark photoperiod cycle as described elsewhere [40]. Eggs laid on moist filter paper were transferred to water basins for hatching. Hatched larvae were reared in well water and fed with grounded laboratory mouse food (Mucedola S.R.L, Milano, Italy). Pupae were transferred to small water containers and placed in screened cages (18 × 18 × 18 cm) for adult emergence. Adult mosquitoes were given 5% sucrose *ad libitum* through soaked cotton pads.

Female BALB/c mice (20 - 25 g) were used as a source of treated blood meals for experimental mosquitoes. Mice were reared in the animal facilities of the University of Camerino, Italy. Rearing and handling of experimental animals were in full compliance with the Italian Legislative Decree on the “protection of animals used for experimental and other scientific purposes” (D. Lgs. 116 of 01/27/92), and in full adherence with the European Directive 2010/63/UE.

### NeemAzal®

NeemAzal® technical grade batch number 052 (Trifolio-M, GmbH, Lahnau, Germany) was used for the experiments. NeemAzal® is a methanolic standardized extract from *Azadirachta indica* seed kernels containing about 55% limonoids. According to the manufacturer, these include 33% azadirachtin A, 16% other azadirachtins (B to K), 4% salannin and 2% nimbin. NeemAzal® also contains 5% fatty acids and other seed kernel components.

A sample of NeemAzal® (1.015 g) taken from the batch number 052 (Trifolio-M, GmbH, Lahnau, Germany) was subjected to column chromatography purification over silica gel (230–400 mesh) using a gradient of eluents of increasing polarity from *n*-hexane/ethyl acetate (EtOAc) 8:2 to EtOAc and then to EtOAc/methanol 1:1. Fourteen fractions (1–14) were thus collected and their composition was analyzed by NMR (on Varian INOVA 500 MHz instrument) and MS (on a LTQ OrbitrapXL Thermo Scientific mass spectrometer) and compared with data published in the literature. This analysis allowed the identification of pure azadirachtin A in fractions 7–8 (eluted with *n*-hexane/EtOAc 4:6), while fraction 6 contained azadirachtin A in mixture with less polar azadirachtins and fraction 9 contained azadirachtin A in mixture with more polar azadirachtins. The amount of azadirachtin A proved to be approximately 520 mg (about 51% of the sample), while the remaining azadirachtins resulted to be approximately 50 mg (about 5% of the sample).

### Preparation and administration of NeemAzal®

A NeemAzal® stock solution was prepared in absolute ethanol, at an azadirachtin A concentration of 50 mg/ml. The stock solution was then diluted to obtain final dosages of 60, 105 and 150 mg of azadirachtin A per kg of mouse body weight. PBS (pH 6.5) with 7.5% Tween 80 and 10% DMSO was used as solvent. Mice were treated intraperitoneally with 100 – 200 μl per animal. Control mice received equal volumes of the solvent solution [36]. For mosquito feeding, mice were narcotized with 100 μl of a 13.1% mixture of xylazine and acepromazine (1:1 ratio) in PBS (pH 7.2).

### Experimental blood meals

Experimental mosquitoes received a total of 5 NeemAzal®-treated blood meals at azadirachtin A doses of 60, 105 and 150 mg/kg, once every 4 days. The study was organized in 3 consecutive experiments, one for each NeemAzal® dose. Four treatment and 4 control group cages containing 150–200 females were used at each NeemAzal® dose, and at each experimental blood meal one treated mouse was exposed to each mosquito cage. Relatively high mosquito survival and feeding rates (80-95%) in both treatment and control groups, allowed to remain with 59 – 81 mosquitoes per cage (4 cages per group) after the fourth blood meal for evaluation of the last experimental feeding event.

Experimental mice were narcotised thirty minutes after the administration of the test solutions, placed on the top of the mosquito cages and exposed to mosquito females for about 1 hour. On the following day, engorged females were counted after cold-immobilization. Males and unfed females were discarded. Gravid females were allowed to lay eggs before the following blood meal.

### Estimation of blood meal size (feeding capacity)

The impact of NeemAzal® on the feeding capacity was estimated by measuring the haematin content of the rectal fluid excreted by the mosquitoes during the blood meal. Briefly, the rectal fluid was collected in Petri dishes (15 cm diameter) positioned in the cages below the feeding mosquitoes. The content of each Petri dish was dissolved in 20 ml of 0.1 M NaOH and incubated overnight, to allow complete conversion of haemoglobin to haematin. To remove blood cell debris, the mixture was spun at 13,000 rpm for 10 minutes [41]. The supernatant containing haematin was serially diluted (6 two-fold dilutions) and aliquots of 200 μl transferred to a 96-well, flat-bottomed microplate (Nunc, Denmark). The absorbance of samples was measured at 392 nm using a FLUOstar Omega spectrophotometer (BMG labtech, Germany). Haemin (Sigma-Aldrich, Italy), was dissolved in 0.1 M NaOH and the haematin solution obtained was used to prepare a calibration curve [42]. The amount of haematin in rectal fluid samples was estimated by extrapolation of the sample’s optical density onto the standard curve. The mean percent reduction in blood meal size was calculated using Abbott’s formula [43] as in the equation below.

$$ \mathrm{Percent}\ \mathrm{reduction}\ \mathrm{in}\ \mathrm{feeding}\ \mathrm{capacity}=\left(1-\frac{\mathrm{Mean}\ \mathrm{haematin}\ \mathrm{content}\ \mathrm{in}\ \mathrm{NeemAza}{\mathrm{l}}^{\circledR}\mathrm{g}\mathrm{roup}\ \left(\upmu \mathrm{g}/\mathrm{mosquito}\right)}{\mathrm{Mean}\ \mathrm{haematin}\ \mathrm{content}\ \mathrm{in}\ \mathrm{control}\ \mathrm{g}\mathrm{roup}\ \left(\upmu \mathrm{g}/\mathrm{mosquito}\right)}\right)\times 100 $$

Means were derived from four replicates (mosquito cages) per group.

### Estimation of oviposition capacity

Two days after each blood meal administration, gravid females were provided with wet filter paper discs for oviposition for 2 subsequent nights [35]. The number of eggs laid on the paper discs were then quantified by imaging analysis, as previously described [44]. The mean percent reduction in oviposition was evaluated using the equation below:

$$ \mathrm{Percent}\ \mathrm{reduction}\ \mathrm{in}\ \mathrm{oviposition}=\left(1-\frac{\mathrm{Mean}\ \mathrm{number}\ \mathrm{of}\ \mathrm{eggs}\ \mathrm{laid}\ \mathrm{in}\ \mathrm{NeemAza}{\mathrm{l}}^{\circledR}\hbox{-} \mathrm{treated}\ \mathrm{group}}{\mathrm{Mean}\ \mathrm{number}\ \mathrm{of}\ \mathrm{eggs}\ \mathrm{laid}\ \mathrm{in}\ \mathrm{control}\ \mathrm{group}}\right)\times 100 $$

Means were derived from four replicates (mosquito cages) per group.

### Assessment of hatchability

To evaluate the impact of NeemAzal® on egg hatchability, 200 eggs per treatment group (50 per replicate) were seeded in 250 ml plastic beakers containing fresh well water. Care was taken to thoroughly mix the eggs on the soaked ovipositor paper discs before sampling. Egg hatching was monitored for a period of 72 hours. This was based on our preliminary findings which showed that, in both control and NeemAzal® treated groups, hatching was completed within 48 hours; beyond this incubation period, no more eggs hatched into larvae. The percent hatchability and the percent reduction in hatchability were evaluated using equations (1) and (2) below, respectively.

1 $$ \mathrm{Percent}\ \mathrm{hatchability}=\frac{\mathrm{Number}\ \mathrm{of}\ \mathrm{larvae}\ \mathrm{hatched}}{\mathrm{Total}\ \mathrm{number}\ \mathrm{of}\ \mathrm{eggs}\ \mathrm{introduced}}\times 100 $$

2 $$ \mathrm{Percent}\ \mathrm{reduction}\ \mathrm{in}\ \mathrm{hatchability}=\left(1-\frac{\mathrm{Number}\ \mathrm{of}\ \mathrm{larvae}\ \mathrm{hatched}\ \mathrm{in}\ \mathrm{NeemAza}{\mathrm{l}}^{\circledR}\hbox{-} \mathrm{treated}\ \mathrm{group}}{\mathrm{Number}\ \mathrm{of}\ \mathrm{larvae}\ \mathrm{hatched}\ \mathrm{in}\ \mathrm{control}\ \mathrm{group}}\right)\times 100 $$

### Data analysis

Data were entered in Excel (Microsoft Co.) and analysed using Excel, Statistical Package for Social Scientists (SPSS) and GraphPad Prism 5 (GraphPad Prism Inc). Comparisons between means of treatment and control groups were performed using Student’s *t*-test. Chi-square test was used to compare group proportions.

## Results

### Effect of repeated NeemAzal® exposure on An. stephensi feeding capacity

During the first 3 blood meals, the average proportion of fed mosquitoes was not different in treated and control groups ranging from 80% to 95% (Table 1). However, a reduction in feeding of about 8% was noted at the fourth and fifth blood meal in the 105 and 150 mg/kg NeemAzal®-treated groups. The proportions of fed *An. stephensi*, in the 105 mg/kg treatment group compared to controls, were reduced by 9% (*p* = 0.0218) and 8% (*p* = 0.0373) at the fourth and fifth blood meal respectively. At the highest treatment dose, the proportion of engorged females was found to be reduced by 7% (p = 0.05) and 8% (*p* = 0.0251) respectively, at the fourth and fifth NeemAzal® exposure (Table 1).

**Table 1:** **Mean numbers and proportions of** ***An. stephensi*** **mosquitoes fed on NeemAzal®-treated and control mice at each of the 5 consecutive blood meals**

**Neem-Azal® dose**	**Blood meal**	**fed mosquitoes in NeemAzal®- treatment groups**		**Fed mosquitoes in control groups**	
		**mean n.** ^**1)**^ **± SD** ^**2)**^	**% (CI**_**95**_ **)** ^**3)**^	**mean n. ±SD**	**% (CI**_**95**_ **)**
150 mg/kg	1	118 ± 7	86 (79–93)	117 ± 14	87 (83–90)
	2	84 ± 11	89 (85–93)	92 ± 15	93 (92–94)
	3	81 ± 11	91 (86–97)	82 ± 14	93 (91–95)
	4	71 ± 9	85 (81–90)	81 ± 17	92 (88–96)
	5	57 ± 8	85 (81–90)	71 ± 18	93 (90–96)
105 mg/kg	1	118 ± 11	82 (76–89)	118 ± 20	82 (75–89)
	2	83 ± 4	92 (90–94)	82 ± 3	93 (92–94)
	3	77 ± 7	90 (83–97)	72 ± 6	90 (87–94)
	4	59 ± 11	81 (70–91)	70 ± 5	90 (86–93)
	5	56 ± 9	82 (75–89)	64 ± 3	90 (86–94)
60 mg/kg	1	139 ± 10	80 (71–89)	117 ± 18	81 (69–93)
	2	108 ± 10	95 (94–97)	81 ± 10	90 (85–94)
	3	86 ± 14	91 (90–92)	72 ± 14	90 (88–92)
	4	73 ± 12	94 (91–96)	63 ± 12	94 (92–97)
	5	59 ± 7	92 (88–96)	54 ± 8	95 (92–97)

^**1)**^mean n. = arithmetic mean number of 4 mosquito replicate cages per treatment group; ^2)^ ± SD = standard deviation; ^**3)**^ CI_95_ = confidence interval at 95%.

**Figure 1:**
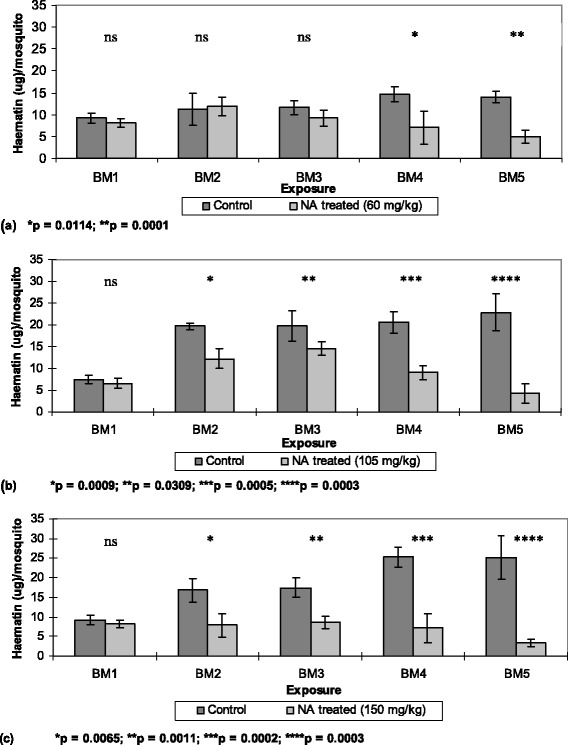
**Blood meal sizes per fed** ***Anopheles stephensi*** **female following treatment with different NeemAzal® doses. (a)** 60 mg/kg, **(b)** 105 mg/kg and **(c)** 150 mg/kg (light bars) or solvent as control (dark bars). BM indicates subsequent blood meals on the same mosquito batches; NA represents NeemAzal®; vertical lines represent 95% confidence interval and ns indicate not significant.

**Figure 2:**
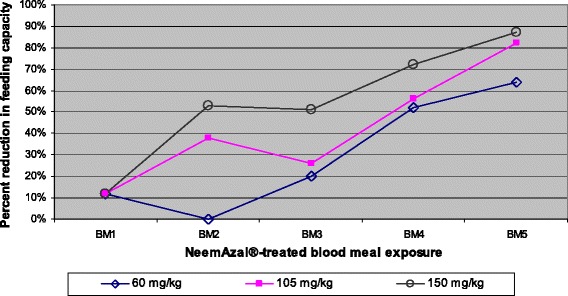
**Dose- and frequency-dependent effect of repeated NeemAzal® treatment on feeding capacity.** BM indicates subsequent blood meals on the same mosquito batches.

Taking into account the number of fed mosquitoes, exposure to repeated NeemAzal®-treated blood meals reduced the mosquito feeding capacity compared to their control counterparts in a dose dependent manner. At a dose of 60 mg/kg azadirachtin A, a reduction of feeding capacity of about 50% was observed after the fourth blood meal (Figures 1a and 2). At 105 mg/kg of azadirachtin A, a significant reduction of 40% in feeding capacity was already obtained after the second blood meal (Figures 1b and 2) and at the highest dose tested (150 mg/kg) the mosquito feeding capacity was reduced by 50% or more from the second blood meal onwards (Figures 1c and 2). At this dose the percent feeding reduction was over 20% higher than that observed in the 60 mg/kg treatment groups.

The extent of feeding capacity of control mosquitoes, as estimated through the haematin quantification in rectal fluid, showed an increasing trend in subsequent blood meals. In particular, a marked increase was observed in the control groups of the 105 and 150 mg/kg dosage experiments from the first to the second blood meal (Figure 1). Such a feeding pattern is characteristic of different mosquito species as previously reported [45].

### Effect of NeemAzal® on oviposition

**Figure 3:**
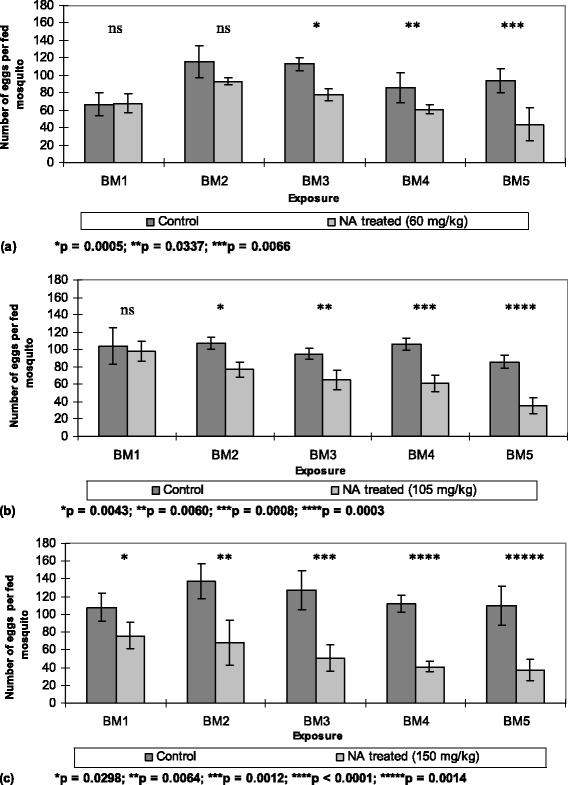
**Effects of different doses of NeemAzal® on oviposition. (a)** 60 mg/kg, **(b)** 105 mg/kg, **(c)** 150 mg/kg (light bars) and solvent control (dark bars). BM indicates subsequent blood meals on the same mosquito batches; NA represents NeemAzal®; vertical lines represent 95% confidence interval; ns indicate not significant.

The administration of NeemAzal® through repeated blood meals also affected oviposition of *An. stephensi* females in a dose-dependent and blood meal frequency-dependent manner (Figure 3).

**Figure 4:**
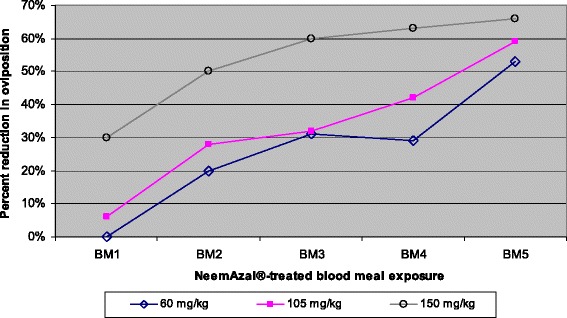
**Dose- and frequency-dependent effect of NeemAzal® treatment on suppression of oviposition.** BM indicates subsequent blood meals on the same mosquito batches

Mosquitoes fed on NeemAzal®-treated blood meals laid significantly less eggs over subsequent blood meals compared to their control counterparts at all tested dosages (Figure 3 and 4). The inhibition in oviposition was most pronounced when mosquitoes fed on mice treated with 150 mg/kg dose of NeemAzal®; at this dosage, after the second blood meal, egg laying was already reduced by about 50% (p = 0.0064) in the treated group compared to their control counterparts. Similar reductions in oviposition of about 60% (p = 0.003) and 50% (p = 0.007) were observed in the 105 mg/kg and 60 mg/kg treatment groups respectively, after the fifth blood meal.

**Figure 5:**
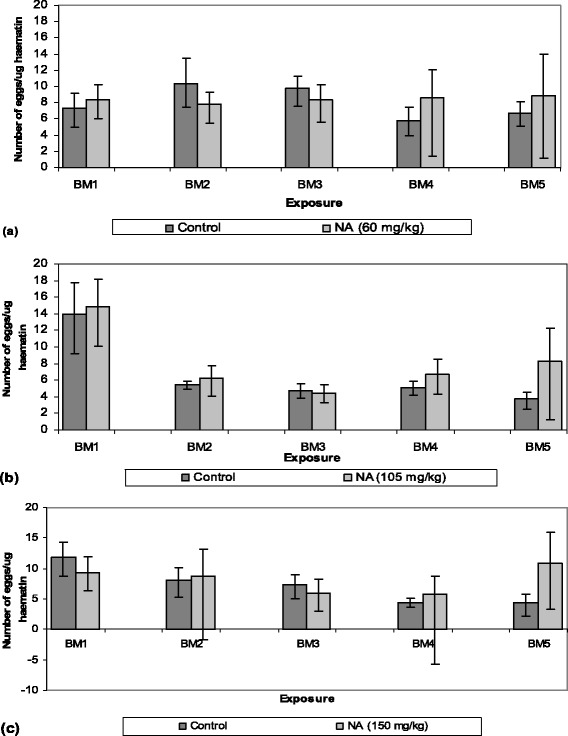
**Estimated amount of eggs produced per μg haematin at different NeemAzal® doses, (a) 60 mg/kg, (b) 105 mg/kg and (c) 150 mg/kg.** BM indicates subsequent blood meals on the same mosquito batches; NA represents NeemAzal®; vertical lines represent 95% confidence interval.

Calculating the number of eggs laid per female mosquito revealed a very similar impact of repeated NeemAzal® treatment on oviposition as observed for feeding. This effect on oviposition appeared to be a consequence of reduced blood intake, given that when the numbers of eggs were calculated per μg of haematin, no evidence of differences emerged between treatment and control females as shown in Figure 5.

### Effect of NeemAzal® on hatchability

**Figure 6:**
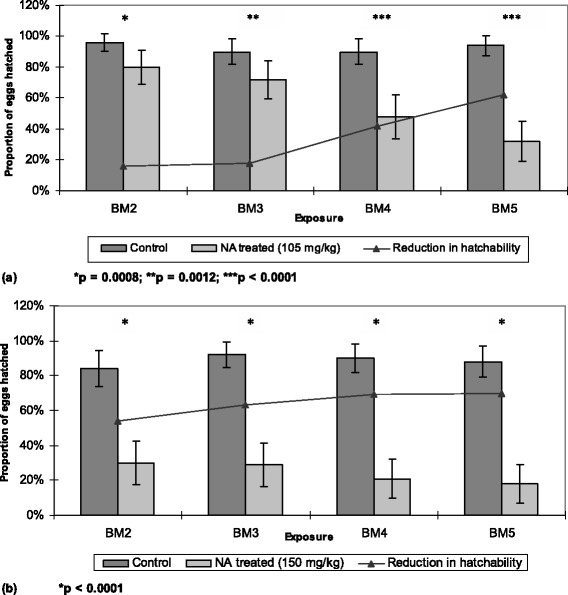
**Proportion of** ***Anopheles stephensi*** **eggs that hatched into larvae at different NeemAzal® doses, (a) 105 mg/kg, (b) 150 mg/kg (light bars) and in control groups (dark bars).** BM indicates subsequent blood meals on the same mosquito batches; NA represents NeemAzal®; vertical lines represent 95% confidence interval.

The fertility of female mosquitoes fed on NeemAzal®-treated BALB/c mice was affected significantly (p < 0.05) at the doses tested, i.e. 105 and 150 mg/kg (Figure 6). At 105 mg/kg, a 16% reduction in egg hatchability (p = 0.0008) was observed after the second blood meal, which gradually increased to 62% (p < 0.0001) after the fifth blood meal. At the 150 mg/kg dose, a 63% hatchability reduction (p < 0.0001) was already observed after the third blood meal, which reached up to 70% (p < 0.0001) after the fifth blood meal.

## Discussion

Neem extracts containing azadirachtin and other bio-active secondary metabolites are used by many communities worldwide in controlling different disease vectors as well as in treatment of malaria and other parasitic diseases. Studies employing direct feeding on neem metabolites or administration through artificial blood meals have shown that insects ingesting azadirachtin and other neem limonoids display reduced fitness due to feeding deterrence, delays in larvae and nymph development, incomplete ecdysis, malformed pupae and adults and reduced fecundity and fertility [30].

In feeding experiments conducted on *Culex tarsalis* and *C. quinquefasciatus*, blood-feeding activity was found to be suppressed when newly emerged adults were fed continuously on 10 parts per million (ppm) or 50 ppm azadirachtin in 10% sucrose solutions for 7 days [33]. Earlier work conducted by our group demonstrated anti-feedant effects of NeemAzal® on *An. stephensi* after administration of the product by membrane feeding of medicated blood or when offered to females in sucrose solution [35]. When females were pre-treated with NeemAzal®-medicated sucrose solution at 100 or 10 μg/ml azadirachtin A equivalents, their blood intake at two subsequent blood meals was significantly reduced. Mosquitoes given NeemAzal® at the same dosages by blood membrane feeding showed reduced feeding at the second but not at the first blood meal. Similarly, in our study reported here, an impact on feeding emerged only from the second blood meal onwards in mosquitoes fed on mice intraperitoneally treated with NeemAzal® at an azadirachtin A dosage of 105 and 150 mg/kg. The fact that comparable secondary anti-feedant effects were found after neem feeding on a treated mammalian host, suggests that azadirachtin, conserves its bioactive molecular conformation and its bioavailability in the mouse, at least briefly.

Calculating the number of eggs laid per female revealed a very similar impact of repeated NeemAzal® treatment on oviposition to that observed for feeding. Egg numbers were significantly reduced in the 150 and 105 mg/kg treatment groups from the first and second blood meal onwards, respectively, and an oviposition reduction of more than 50% was observed after the fifth blood meal at all the tested dosages. The effect on oviposition appeared to be mainly a consequence of reduced blood intake, since no difference was observed between treatment and control females with regards to the numbers of eggs per μg of haematin excreted in the rectal fluid of blood sucking mosquitoes, which was used as a feeding estimate. In our previous study, whereby NeemAzal® was administered through membrane feeding, a significant reduction in the numbers of eggs per μl of blood was recorded in the 100 μg/ml group after the second blood meal, but no oogenesis inhibitory effect on oviposition was found in mosquitoes treated with NeemAzal® medicated sucrose solution [35].

For comparison, in *C. tarsalis* and *C. quinquefasciatus* the oviposition rate and size of egg raft was found to be reduced when females were fed on sucrose solutions containing 10 ppm azadirachtin before blood feeding [33]. Studying the azadirachtin effects on the haematophagous vector of chagas desease *Rhodnius prolixus*, Garcia and colleagues (1990) observed a strong reduction of the number of eggs laid by females after administration of 1 and 5 μg/ml azadirachtin by membrane feeding [46]. By administering 3 consecutive plain blood meals after the treated one, the effect was found to be irreversible in the 5 μg/ml group and only partially reversible at 1 μg/ml. The authors hypothesized that small amounts of azadirachtin may stably bind to various insect organs including brain, prothoracic glands and ovaries and thus interfere with endocrine regulation [46,47]. An irreversible impact on vitellogenesis was also observed by Dhar and colleagues [48], studying the effects of long-term exposure to neem volatiles on *An. stephensi* and *An culicifacies* females. From this study it emerged that neem components can exert biological effects also after absorption through the cuticle or passage through spiracles (Dhar 1996).

Morphological alterations of the ovaries were reported and discussed in our earlier publications on azadirachtin A effects in *An. stephensi* [35]. The ultrastructural investigations on ovaries from NeemAzal®-treated females revealed distinct structural modifications indicative of a complete block of oogenesis, impairment of vitellogenesis, vitelline envelope formation, and degeneration of follicle cells. Morphologically altered ovarioles were recorded next to structurally normal ones. The results of the ultrastructural study suggested that NeemAzal® impairs hormone control of oogenesis and exerts a cytotoxic effect on both follicular cells and oocytes [35]. These findings are in line with earlier observations in other insects suggestive of detrimental effects of azadirachtin A on the neurohormone producing gland [49], reproductive tissue [50] as well as on gut epithelial cells [51]. On the basis of the discussed mode of azadirachtin A action, the impact of NeemAzal® on egg hatchability recorded in this study was not an unexpected finding. The proportion of eggs that were able to hatch was reduced in the 105 and 150 mg/kg treatment group from the second blood meal onwards. At these dosages hatchability was inhibited by more than 60% and 70%, respectively, after the fifth blood meal taken by the females on NeemAzal®-treated mice.

In our study, the survival of mosquitoes appeared not to be affected by the repeated NeemAzal® administration even at the highest tested dosage (data not shown). This finding is in line with other azadirachtin studies that showed a moderate impact on insect longevity in repeated exposure schemes [33,46]. Azadirachtin exerts cytotoxic effects at high concentrations; however, in *Rodnius prolixus* and *Locusta migratoria* the compound was found to be rapidly metabolized and excreted in the form of dihydroazadirachtin although small amounts could be retrieved in the head and visceral tissues weeks after its application [52,53].

Taken together, these findings are very encouraging and warrant further studies in human hosts consuming neem-based preparations for various purposes. These natural products are eco-friendly and not toxic to humans and other vertebrate hosts according to studies conducted by Al Sharook [54]. If scientifically validated and produced as standardized phytomedicines may play a prominent role in malaria control programmes of endemic regions. In Nigeria for instance, a standardised neem leaf extract traded as IRACARP® is being used in the management of patients co-infected with malaria and HIV [55]. In Kenya, prophylactic neem extracts are being marketed as Neem herbal tea, a nutraceutical product that provides a minimum of 22 mg/kg neem leave extracts per tea bag [56]. The use of standardised phytomedicines in situations where access to antimalarial drugs is hampered or delayed might save lives, particularly in children who are at high risk of rapidly developing severe forms of the disease [25].

All the above observations indicate the need for field trials to assess the curative efficacy and safety of neem-based products already in use. In areas where members of communities regularly use neem during high transmission seasons to prevent malaria episodes, it would be highly interesting to assess and dissect the prophylactic and transmission blocking efficacy of such traditional remedies at a community level.

## Conclusions

The diffused use of neem in the treatment of malaria and its well known insect growth regulatory properties guided this study of the multi-factorial effects of this plant extract on the mosquito vector fitness *in vivo* using the mouse as the vertebrate host and *An. stephensi* as the vector. Our results show that repeated exposure to NeemAzal®-treated blood meals can result in a cascade of events leading to a significant reduction in the fitness of *An. stephensi*. The mosquito feeding capacity, oviposition and egg hatchability are all decreased in a dose and blood meal frequency dependent manner. In a hypothetical field situation of a community regularly consuming neem treatments and thus exposing the *Anopheles* vectors repeatedly to the bioactive components, cumulative effects as demonstrated here for the product NeemAzal® may potentially block the transmission of malaria parasites by disrupting the vector fitness and, consequently, vectorial capacity.

The findings from this study and previous work highlight the challenging prospect of designing formulations on the basis of neem plant parts rich in azadirachtin A as multi-target herbal medicines exhibiting curative, preventive, transmission-blocking and anti-vectorial properties.
